# Intestinal tuberculosis masquerading as carcinoma colon: a case report of diagnostic quandary in low-resource setting

**DOI:** 10.1093/jscr/rjac210

**Published:** 2022-05-24

**Authors:** Sagar Panthi, Pradeep Khatiwada, Seema Adhikari, Rochana Acharya, Durga Neupane, Ananta Sharma, Pramodman Singh Yadav, Padmini Yadav, Raksha Bhattarai, Bhawani Khanal

**Affiliations:** Department of General Surgery, B. P. Koirala Institute of Health Sciences, Dharan, Province 1, Nepal; Department of General Surgery, B. P. Koirala Institute of Health Sciences, Dharan, Province 1, Nepal; Department of General Surgery, B. P. Koirala Institute of Health Sciences, Dharan, Province 1, Nepal; Department of General Surgery, B. P. Koirala Institute of Health Sciences, Dharan, Province 1, Nepal; Department of General Surgery, B. P. Koirala Institute of Health Sciences, Dharan, Province 1, Nepal; Department of General Surgery, B. P. Koirala Institute of Health Sciences, Dharan, Province 1, Nepal; Department of General Surgery, B. P. Koirala Institute of Health Sciences, Dharan, Province 1, Nepal; Department of General Surgery, B. P. Koirala Institute of Health Sciences, Dharan, Province 1, Nepal; Department of General Surgery, B. P. Koirala Institute of Health Sciences, Dharan, Province 1, Nepal; Department of General Surgery, B. P. Koirala Institute of Health Sciences, Dharan, Province 1, Nepal

## Abstract

Intestinal tuberculosis (TB) is a rare condition comprising a majority of the extra-pulmonary TB cases. Owing to a similar clinical presentation, ultrasonographic and biopsy findings of intestinal TB with that of other abdominal pathologies such as carcinoma colon, their clinical delineation is very difficult unless aided with other modalities of investigations such as colonoscopy, culture of the biopsy material, etc. and even advanced methods such as polymerase chain reaction and gene X-pert of the biopsy material. Having all these investigations may not even lead to a correct diagnosis of intestinal TB as evidenced in the reported cases in the literature, advocating the need of diagnostic laparoscopy in the diagnosis of intestinal TB to eliminate extensive and unnecessary surgeries. Here, we present a case of intestinal TB in a 51-year-gentleman who got diagnosed in the course of treatment for a suspected carcinoma colon.

## INTRODUCTION

Intestinal tuberculosis (TB) is a rare condition but encompasses a majority of the cases of extra-pulmonary TB. As it shares similar clinical features, ultrasonographic (USG) and biopsy findings, it is difficult to delineate from other abdominal conditions including carcinoma colon. The recent developments in colonoscopy, biopsies and acid-fast bacilli (AFB) smears can enable an early diagnosis [1]. In this case study, we report a case of intestinal TB that got diagnosed in the course of treatment for suspected carcinoma colon.

## CASE REPORT

**Figure 1:**
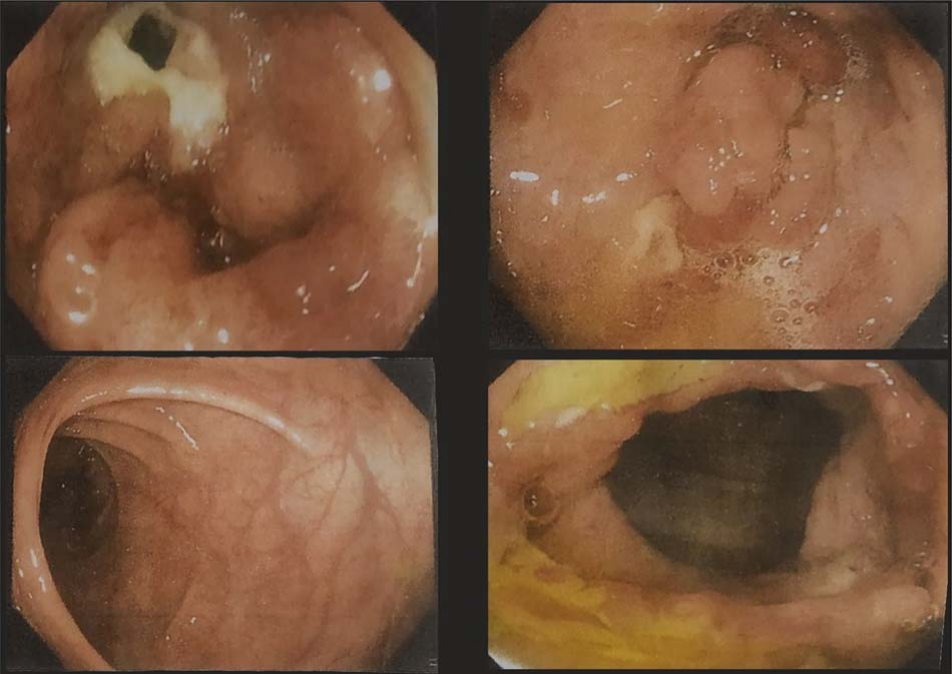
Colonoscopy images showing ulcero-proliferative growth in the cecum and descending sigmoid junction.

**Figure 2:**
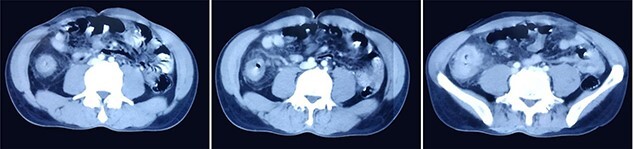
CT images showing segmental asymmetrical circumferential mass-like wall thickening of the cecum and ascending colon over the length of 7.6 cm causing luminal narrowing with enhancing soft tissue extension into the adjacent pericolic fat with loss of fat plane with the right psoas major muscle, pericolic fat stranding along with thickening of adjacent peritoneal lining.

A 51-year-gentlemen and a smoker visited our institute with a history of abdominal pain, predominantly over the umbilical area, constant in nature, associated with anorexia, back pain and occasional chest pain for about 1 year. Besides pallor, other examination findings were grossly normal. His body mass index was 17 mg/m^2^. The comprehensive blood panel, renal function test and liver function test yielded normal findings. USG of abdomen and pelvis showed thickened cecum and terminal ileum with few adjacent mesenteric lymph nodes likely to be an infective/inflammatory pathology along with coexisting right nephrolithiasis. Intestinal TB was suspected and further investigations were done but reported negative Mantoux test, AFB smear and normal chest X-ray findings. Colonoscopy showed ulcero-proliferative growth in the cecum and descending sigmoid junction with punch biopsy from the cecal growth showing mitotic lesion suggestive of carcinoma colon (Fig. 1). A repeat colonoscopic biopsy of the cecal growth suggested diffuse active colitis. Computed tomography (CT) revealed segmental asymmetrical circumferential mass-like wall thickening of the cecum and ascending colon over the length of 7.6 cm causing luminal narrowing with enhancing soft tissue extension into the adjacent pericolic fat with loss of fat plane with the right psoas major muscle, pericolic fat stranding along with thickening of adjacent peritoneal lining (Fig. 2). The presence of multiple homogeneously enhancing pericolic, ileocolic, superior mesenteric artery and para-aortic groups of lymph nodes suggested colon carcinoma (T4aN2b). It also revealed a small iso-dense lesion showing homogeneous enhancement on the arterial phase in segment VII of the right lobe of the liver abutting capsule suggestive of metastasis. Carcinoembryonic antigen (CEA) was found to be slightly raised (7 ng/dl).

In suspicion of the carcinoma colon, he underwent total colectomy under general anesthesia. The operative findings included large ulcero-proliferative lesion of size 5 × 4 cm at cecum and single polypoidal lesion of size 2 × 2 cm at descending colon and sigmoid colon junction. Liver and spleen were found to be normal. There was no ascites, no peritoneal or omental deposits or tubercles that could suggest intestinal TB. Following the postoperative phase, patient followed up after 4 weeks with the histopathological report of the colectomy specimen that reported the features suggestive of necrotizing granulomatous lymphadenitis, possibly intestinal TB with no any evidence of carcinoma colon. Owing to the histopathological diagnosis, the patient was then started on anti-tubercular therapy with a regular follow-up. He has now completed the anti-tubercular therapy and doing well with his life.

## DISCUSSION

Intestinal TB is one of the common entities among the spectrum of extra-pulmonary TB, the most common sites of involvement being the terminal ileum and cecum [2]. On gross examination, apart from typical tuberculous lesions in the form of transverse ulcers, strictures, hyperplastic lesions and serosal tubercles, intestinal perforation and ischemic bowel are identified too [3]. The diagnosis of the extra-pulmonary TB remains challenging due to the inconspicuous endoscopic and radiographic findings [4].

Although the Mantoux test is considered to be sensitive for TB, a negative Mantoux test, AFB smear and normal chest X-ray misled to the diagnosis in our case. A study conducted in Malaysia showed the sensitivity of Mantoux test in active TB as 86% [5]. The positive predictive value of the Mantoux tests is around 20%, whereas the negative predictive value is 95.4% [6]. Negative Mantoux test in immunocompetent individuals are seldom questioned due to its high negative predictive value. Culture of biopsy material remains the gold standard for the diagnosis of intestinal TB but usually requires 4–6 weeks until results are obtained [7, 8]. Furthermore, studies in patients with colonic TB indicate that positive culture can be found in only one-third of patients or even less [9, 10]. Although the advanced investigations like polymerase chain reaction and Gene X-pert play a promising role in the diagnosis of pulmonary TB, it has shown poor sensitivity and specificity for the detection of abdominal TB from ascitic fluid samples [11] compared with sputum [12] and cerebrospinal fluid [13].

Isolated colonic TB is rare with only 2–3% cases of abdominal TB have isolated colonic involvement. Nonspecific symptoms often mimic Crohn’s disease and malignancy, along with colonoscopy and biopsy findings. The distinction may be impossible without surgical resection. The combined yield of biopsy and AFB smear has improved from 75% in earlier studies to 92% in a recent study [14]. Diagnostic laparoscopy is currently emerging as a valuable diagnostic tool, but misdiagnosis can still be a possibility.

In a study by Rath *et al.* [15], a large percentage of patients with colonic TB histology revealed only chronic nonspecific changes in the form of chronic inflammatory cells in the lamina propria. Preoperative colonoscopic biopsy of our case also showed nonspecific finding of diffuse active colitis. Though raised CEA level supported colorectal carcinoma in our case, a study by Polat *et al.* showed that the CEA measurement in the diagnosis of colorectal cancer patients at all disease stages had 51.9% sensitivity at 90% specificity for a cut-off level 2.41 ng/ml. This improved significantly upon coupling with CA 19-9 biomarker for diagnosing distal metastasis and lymph node invasion [16]. Patients with peritoneal TB and TB empyema are also reported to have higher level of CEA [17, 18].

This case thus highlights the limitations in the available method of investigations pertaining to its sensitivity, specificity and reliability of the test. In the low-resource setting among the vulnerable population, it is essential to combine all the clinical examinations, endoscopic and histopathologic findings despite the presence of signs of carcinoma, to rule out clinical suspicion of TB, eliminating the need of extensive and unnecessary surgeries.

## CONCLUSIONS

The diagnosis of intestinal TB is very much difficult in a low-resource setting owing to the low sensitivity, specificity and reliability of the available tests for TB and similar presentations with those of other abdominal pathologies such as carcinoma colon, Crohn’s disease, etc., thus advocating the need of combining diagnostic laparoscopy with the clinical examinations, endoscopic and histopathologic findings to narrow down the clinical suspicion of TB eliminating the need of extensive and unnecessary surgeries.

## CONFLICT OF INTEREST STATEMENT

None declared.

## FUNDING

None.
